# Mycotoxin Removal and Transcriptional Response of *Pichia fermentans* KCB21_L2

**DOI:** 10.3390/foods14244181

**Published:** 2025-12-05

**Authors:** Carolina Gómez-Albarrán, Silvia Rodríguez-Pires, Alba Sáez-Matía, Carlos Luz, Belén Patiño, Jéssica Gil-Serna

**Affiliations:** 1 Department of Genetics, Physiology and Microbiology, Faculty of Biological Sciences, University Complutense of Madrid, 28040 Madrid, Spain; caroli13@ucm.es (C.G.-A.); silvia.rodriguez.pires@cib.csic.es (S.R.-P.); jgilsern@ucm.es (J.G.-S.); 2 Proteomics and Genomics Facility, Centro de Investigaciones Biológicas Margarita Salas (CIB), Consejo Superior de Investigaciones Científicas, 28040 Madrid, Spain; 3 Department of Cellular and Molecular Biology, Centro de Investigaciones Biológicas Margarita Salas (CIB), Consejo Superior de Investigaciones Científicas, 28040 Madrid, Spain; 4 Laboratory of Food Chemistry and Toxicology, Faculty of Pharmacy, University of Valencia, 46100 Burjassot, Spain; carlos.luz@uv.es

**Keywords:** RNAseq, biological removal, yeasts, mycotoxins, adsorption

## Abstract

The presence of mycotoxins in food poses a significant risk to food safety, and it is essential to develop effective and safe detoxification strategies. In this study, we demonstrate the strong ability of *Pichia fermentans* KCB21_L2, a yeast isolated from kefir, to eliminate aflatoxin B_1_, fumonisin B_1_ and ocratoxin A. Viable cells removed aflatoxin B_1_ and fumonisin B_1_ more efficiently than heat-inactivated cells, particularly at pH values of 5.5 and 7.0, suggesting the involvement of an active removal process. Subsequently, we evaluated the capacity of *P. fermentans* KCB21_L2 to remove mycotoxins at high concentrations and investigated the underlying molecular and cellular responses. The yeast effectively eliminated high levels of all three mycotoxins. Transcriptional analysis revealed the activation of metabolic pathways related to amino acid catabolism and fatty acid metabolism, likely reflecting an adaptive stress response. However, no significant upregulation of specific genes related to mycotoxin-degrading enzymes was observed. In conclusion, the reduction process may involve multiple factors, including stress response pathways, possible production of organic acids, adsorption of mycotoxins into the cell wall, and constitutively expressed enzymes capable of degrading mycotoxins. In general, these findings highlight the multifactorial nature of yeast-mediated mycotoxin removal and establish *P. fermentans* KCB21_L2 as a promising candidate for safe biological decontamination in food systems.

## 1. Introduction

Mycotoxins are secondary metabolites produced by fungi such as *Aspergillus* spp., *Penicillium* spp., *Alternaria* spp., and *Fusarium* spp. To date, more than 700 mycotoxins have been identified, among which aflatoxin B_1_ (AFB_1_), fumonisin B1 (FB_1_), and ochratoxin A (OTA) are extremely relevant due to their significant impact on human and animal health [1,2]. AFB_1_ is considered a Group 1 human carcinogen by the International Agency for Research on Cancer (IARC), since chronic exposure to it has carcinogenic, teratogenic, and mutagenic effects, primarily targeting the liver [3,4,5]. In contrast, acute exposure to FB_1_ typically results in gastrointestinal symptoms, whereas chronic toxicity has been associated with hepatotoxicity and carcinogenesis in the esophagus [6]. The toxicity of OTA is related to its nephrotoxic, hepatotoxic, teratogenic, immunotoxic, neurotoxic, and genotoxic properties, as well as its ability to produce kidney and liver tumors in animals [7,8]. Consequently, both FB_1_ and OTA have been classified by the IARC as possibly carcinogenic to humans (Group 2B).

The presence of mycotoxins in food also contributes to significant economic losses in the agrifood sector. According to Eskola et al. [9], between 60% and 80% of global crops and staple foods may be contaminated with mycotoxins. In addition, these compounds can occur in any stage of the food chain and resist most of the common treatments applied to foods, which makes their removal particularly challenging [10,11]. Therefore, it is essential to develop strategies aimed at reducing or eliminating mycotoxins present in food products [12]. Although physical and chemical methods for mycotoxin decontamination are available, their application is limited because they can increase costs, modify natural flavor, reduce nutritional value, etc. [13]. Biological decontamination is considered the safest method from environmental and nutritional perspectives, as it preserves the organoleptic properties of food while offering high specificity and efficiency. This approach involves the use of microorganisms (bacteria, yeasts, or filamentous fungi) or their enzymes to degrade, transform, or bind mycotoxins, thus preventing the harmful effects of consuming contaminated food [13,14].

Biological decontamination requires that both the microorganisms used, and their degradation products (if any) are harmless [15]. For this reason, microorganisms isolated from fermented foods are promising candidates as biological decontaminating agents due to their well-established safety profiles [16,17]. Kefir is a fermented dairy product produced through the lactic and alcoholic fermentation of milk via kefir grains, which includes a complex symbiotic community of lactic acid bacteria, acetic acid bacteria, and yeasts [18,19]. Some research has reported kefir’s ability to eliminate mycotoxins such as AFB_1_ and OTA [20,21,22]. Nevertheless, few studies have systematically assessed the decontamination potential of individual species within kefir. Among these, *Pichia fermentans* is one of the most prevalent yeasts and plays a key role in the formation of the biofilm that supports the initial development of kefir grains [23,24,25,26,27].

A critical step in the application of microorganisms as mycotoxin decontamination agents is to elucidate the mechanism underlying their activity [28]. In recent years, omics-based approaches, particularly transcriptomics, have emerged as powerful tools for uncovering the molecular and physiological pathways involved in mycotoxin removal [29,30]. Therefore, in this work, we aimed to assess the ability of the kefir-isolated yeast *P. fermentans* KCB21_L2 to eliminate AFB_1_, FB_1_, and OTA, evaluating the effect of pH and physiological state on this process. In addition, we aimed to elucidate the molecular and biological mechanisms underlying the response of *P. fermentans* KCB21_L2 to exposure to these mycotoxins through transcriptomic analysis.

## 2. Materials and Methods

### 2.1. Isolation and Identification of Pichia fermentans Isolated from Kefir

One gram of kefir was serially diluted in 0.9% (*w*/*v*) NaCl, plated onto Rose Bengal Chloramphenicol Agar (Condalab, Madrid, Spain), and incubated for 48 h at 30 °C. Subsequently, yeast isolates were purified on Potato Dextrose Agar (PDA) plates (Condalab, Spain) using the streak plate method and stored in 15% glycerol (Fisher Chemical, Loughborough, UK) at −80 °C until required.

The isolated yeast was identified by sequencing the D1–D2 region of the 26S rRNA gene. Firstly, a direct colony PCR was performed to amplify this region using the primer pair NL1 (5′-GCATATCAATAAGCGGAGGAAAAG-3′) and NL4 (5′-GGTCCGTGTTTCAAGACGG-3′), following the protocol described by Kurtzman and Robnett [31] with the addition of an initial 10 min denaturation step. PCR assays were performed using an Eppendorf Mastercycler Nexus^®^ thermocycler (Eppendorf, Hamburg, Germany). Each reaction mixture contained a colony picked with sterile tips, 1 µL of each primer (20 µM) (Metabion, Planneg, Germany), 12.5 µL of NZYTaq II 2× Green Master Mix (NZYTech, Lisbon, Portugal), and 10.5 µL of molecular-biology-grade water (PanReac AppliChem, Barcelona, Spain). Three reactions were performed to obtain enough PCR products for sequencing, which were visualized in 1.5% agarose gel electrophoresis (Condalab, Madrid, Spain) using 1× TAE buffer (Tris-acetate 40 mM and EDTA 1.0 mM) and 3 μL of Green Safe Premium (1 μg/mL) (NZYTech, Lisbon, Portugal). The NZY Ladder V (NZYTech, Lisbon, Portugal) was used as a molecular size marker. Electrophoresis was performed at 80 V for 30 min and then visualized under UV light (ETX-20-M, Vilber Lourmat, Paris, France).

The PCR products were purified using the NZYGelpure Kit (NZYTech, Portugal) and sequenced in both directions at Macrogen facilities (Madrid, Spain) using an ABI PRISM 3730XL DNA sequencer (Applied Biosystems, Foster City, CA, USA) following the manufacturer’s instructions. The sequences were assembled with UGENE v33.0 and the resulting consensus sequences were compared against the NCBI nucleotide database using BLAST to obtain species-level identification.

### 2.2. Study of the Ability of Pichia fermentans KCB21_L2 to Remove Aflatoxin B_1_, Fumonisin B_1_, and Ochratoxin A

#### 2.2.1. Removal Assays Using Viable and Heat-Inactivated Cells

*P. fermentans* KCB21_L2 suspensions were prepared from 24 h old cultures on PDA plates at 30 °C. Cells were harvested in 0.9% (*w*/*v*) NaCl solution, and cell concentration was determined using a Thoma counting chamber (Marienfeld, Lauda-Königshofen, Germany). Finally, the suspension was adjusted to 5 × 10^7^ cells/mL. These resulting suspensions constituted viable cells (VCs). Stock solutions of AFB_1_, FB_1_, and OTA (Sigma-Aldrich, Darmstadt, Germany) were prepared in methanol to a final concentration of 1,000,000 μg/L. These solutions were diluted to the final concentrations specified in each experiment.

The first removal assays of AFB_1_, FB_1_, and OTA using *P. fermentans* KCB21_L2 were performed in 96-well polystyrene microplates (Corning™, New York, NY, USA) at three pH levels (3.0, 5.5, and 7.0) and under two cell conditions: viable cells (VC) and heat-inactivated cells (HIC). The pH values were adjusted using HCl (0.5 M) or NaOH (0.5 M), depending on whether a more acidic (pH 3.0) or neutral (pH 7.0) condition was required. The final concentrations of the mycotoxins were 10 μg/L AFB_1_, 100 μg/L FB_1_, and 0.5 μg/L OTA, respectively. The experimental protocol for both VC and HIC assays followed the procedure described by Gómez-Albarrán et al. [32]. All samples and controls were analyzed in duplicate. Microplates were incubated for 48 h at 30 °C, and yeast growth was monitored by measuring turbidity at 630 nm using a plate reader (Dutscher, Bernolsheim, France). Samples were filtered through 0.22 μm filters (Fisherbrand, Thermo Fisher Scientific, Madrid, Spain), evaporated using a rotary vacuum concentrator (Eppendorf™ Concentrator Plus with Pump and GB Plug, Hamburg, Germany), and resuspended in 1× TAE buffer to minimize the influence of culture medium and pH on further analysis. The resulting extracts were stored at −20 °C until Enzyme-Linked Immunosorbent Assay (ELISA) quantification.

#### 2.2.2. Removal Assays at High Mycotoxin Concentrations

The assays were performed in pre-sterilized flasks using 2 mL of *P. fermentans* KCB21_L2 suspension in 18 mL of PDB (pH of 3.0, 5.5, or 7.0) with 200 μL of AFB_1_, FB_1_, or OTA. Three final concentrations were tested for each mycotoxin: 20, 100, and 200 μg/L for AFB_1_; 200, 1000, and 2000 μg/L for FB_1_; and 1, 10, and 100 μg/L for OTA. Growth control conditions included cultures of *P. fermentans* KCB21_L2 in the absence of mycotoxins but including methanol. All conditions were tested in duplicate, and samples were incubated at 30 °C in an orbital shaker at 100 rpm for 48 h. Those containing mycotoxins were subsequently filtered through 0.22 μm filters, evaporated, and resuspended in 1× TAE buffer. The resulting preparations were stored at −20 °C until ELISA quantification.

### 2.3. Transcriptome Sequencing of Pichia fermentans KCB21_L2

#### 2.3.1. RNA Extraction

First, a 2 mL suspension of *P. fermentans* KCB21_L2 (5 × 10^7^ cells/mL) was inoculated into a flask containing 18 mL of Potato Dextrose Broth (PDB, pH 5.5) (Condalab, Madrid, Spain) supplemented with AFB_1_, FB_1_, and OTA extracts up to final concentrations of 200 μg/L AFB_1_, 1000 μg/L FB_1_, and 100 μg/L OTA. In control samples, the volume of mycotoxin extracts was replaced with methanol (solvent). All conditions were evaluated in triplicate, and samples were incubated at 30 °C in an orbital shaker at 100 rpm for 12 h. After incubation, cultures were centrifuged at 2500× *g* for 10 min to collect the cells for RNA extraction. Supernatant was filtered through 0.22 μm filters and stored at −20 °C for subsequent analysis by Ultra-High-Performance Liquid Chromatography coupled with Quadrupole Time-of-Flight Mass Spectrometry (UHPLC-Q-TOF-MS) to assess the possible presence of degradation products associated with AFB_1_, FB_1_, and OTA.

Total RNA was extracted from yeast cells after enzymatic treatment with lyticase using the RNeasy Mini Kit (QIAgen, Hilden, Germany), following the manufacturer’s instructions. To remove genomic DNA, samples were treated with the RNase-Free DNase Set Kit (QIAgen, Hilden, Germany) for 15 min. RNA quality was verified by electrophoresis on a 2% agarose gel (Condalab, Madrid, Spain) using the same conditions as those described in Section 2.1. RNA concentration was measured using a Nanodrop ND 1000 spectrophotometer (Thermo Scientific, Waltham, MA, USA). Samples were normalized to 1 μg/μL and stored at −80 °C until shipment to Macrogen facilities (Macrogen Inc., Seoul, Republic of Korea). All samples presented an RNA integrity number (RIN) greater than 6. Libraries were prepared using the TruSeq Stranded mRNA kit (Illumina, Foster City, CA, USA) according to the manufacturer’s instructions, and sequencing was performed using Illumina technology and the NovaSeq 6000 150 PE platform (2 × 150 bp; 6 Gb/sample).

#### 2.3.2. Bioinformatic Analysis of Transcriptome Data

The quality of raw reads from RNA sequencing was assessed using FastQC v0.12.0 [33], and adapters and low-quality sequences were removed with TrimGalore v0.6.5 [34]. Next, reads were mapped to the *P. fermentans* CDLB.YE03 reference genome (Gen-Bank: GCA_035610515.1) using HISAT2 v2.0.4 [35]. The genome was previously structurally annotated using Augustus [36] with the pre-trained set for *Pichia stipitis* as the model species. All analyses were performed, and graphs were constructed using R version 4.4.1. Principal Component Analysis (PCA) was performed on the normalized reads with the DESeq2 ‘rld’ function and visualized with the ‘plotPCA’ function. Subsequently, differential expression analysis was carried out with the Bioconductor DESeq2 package v1.44.0 [37]. For this analysis, the raw data were first transformed using a regularized logarithmic transformation (rlog), which stabilized the variance and facilitated the interpretation of the differences between samples. The samples were compared in pairs by calculating the relative change in expression (log_2_ fold change, log_2_FC). Genes with an adjusted *p*-value < 0.05 and |log_2_FC| ≥ 2 were considered differentially expressed genes (DEGs). Those with higher expression compared to the control (log_2_FC ≥ 2) were classified as overexpressed or upregulated genes, whereas those with lower expression (log_2_FC ≤ −2) were considered repressed or downregulated genes.

DEGs for each mycotoxin treatment were visualized using volcano plots generated with the EnhancedVolcano package v1.22.0 in R. In addition, common DEGs across the three conditions (AFB_1_, FB_1_, and OTA) were represented using bar charts and UpSet plots. All DEGs were functionally annotated with eggnog v2.1.12, PANNZER2 (Protein ANNotation with Z-scoRE), dbCAN 2 (for the identification of CAZymes), Protein family (Pfam), Clusters of Orthologous Groups (COG), Gene Ontology (GO), and the Kyoto Encyclopedia of Genes and Genomes (KEGG).

### 2.4. Quantification of Mycotoxins and Their Degradation Products

#### 2.4.1. Enzyme-Linked Immunosorbent Assay (ELISA)

AFB_1_, FB_1_, and OTA were quantified using RIDASCREEN^®^ Aflatoxin B1 30/15 Art. No. R1211, RIDASCREEN^®^ Fumonisin Art. No. R3401, and RIDASCREEN^®^ Ochratoxin A 30/15 Art. No. R1311 (R-Biopharm, Darmstadt, Germany), respectively, following the manufacturer’s instructions. The colorimetric reaction was measured at 450 nm using a microplate reader (Dutscher, Bernolsheim, France). A six-point calibration curve was prepared for AFB_1_ (0; 1; 5; 10; 20; 50 μg/L) (% (B/B0) = −37.029 log(concentration) + 71.761; R^2^ = 0.968), FB_1_ (0; 0.025; 0.074; 0.222; 0.666; 2 mg/L) (% (B/B0) = −38.856 log(concentration) + 21.541; R^2^ = 0.964) and OTA (0; 0.03; 0.1; 0.3; 1; 3 μg/L) (% (B/B0) = −43.832 log(concentration) + 20.671; R^2^ = 0.964), as provided in each RIDASCREEN^®^ ELISA kit. The percentage of absorbance (%(B/B0)) was calculated using the formula:

$$\%\left(B/B0\right)=\frac{\left(B\right)}{\left(B0\right)}\times 100$$

where B represents the absorbance of the standard or sample, and B0 the absorbance of the blank. The absorbance data obtained from the removal tests were interpolated using the standard curve to determine the concentrations of AFB_1_, FB_1_, and OTA remaining after incubation. Standard curves were generated using Microsoft Excel^®^ (Microsoft Corporation, Washington, DC, USA).

#### 2.4.2. Ultra-High-Performance Liquid Chromatography Coupled with Quadrupole Time-of-Flight Mass Spectrometry (UHPLC-Q-TOF-MS)

Supernatants were diluted with MilliQ water, filtered with 0.22 µm and analyzed using an UHPLC (1290 Infinity LC, Agilent Technologies, Santa Clara, CA, USA) coupled with a quadrupole time of flight mass spectrometer (Agilent 6546 LC/Q-TOF, Agilent Technologies, Santa Clara, CA, USA) operating in positive and negative ionization mode. Chromatographic separation was performed with an Agilent Zorbax RRHD SB-C18, 2.1 × 50 mm, 1.8 µm column. Mobile phase A was composed of Milli-Q water, and acetonitrile was used for mobile phase B (both phases were acidified with 0.1% formic acid), with gradient elution, as follows: 0 min, 2% B; 22 min 95% B; 25 min, 5% B. The column was equilibrated for 3 min before every analysis. The flow rate was 0.4 mL/min, and 5 µL of sample was injected.

Dual AJS ESI source conditions were as follows: gas temperature: 325 °C; gas flow: 10 L/min; nebulizer pressure: 40 psig; sheath gas temperature: 295 °C; sheath gas flow: 12 L/min; capillary voltage: 4000 V; nozzle voltage: 500 V; Fragmentor: 120 V; skimmer: 70 V; product ion scan range: 100–1500 Da; MS scan rate: 5 spectra/s; MS/MS scan rate: 3 spectra/s; maximum precursors per cycle: 2; and collision energy: 10, 20, 40 eV. The analysis of the metabolites was carried out in triplicate. Integration, data elaboration, and identification of metabolites were managed using MassHunter Qualitative Analysis software B.08.00 and library PCDL Manager B.08.00. Specific libraries were used for the identification of mycotoxins and their degradation products of AFB_1_ [38], FB_1_ [39] and OTA [40]. The molecular formula of the degradation products included in the study can be seen in Supplementary Materials (Tables S1–S3).

A score value of 95% and a Delta mass error of 1 ppm were used as confidence intervals. For the calibration of the UHPLC-Q-TOF-MS analysis method, commercial standards for the AFB_1_ [38], FB_1_ [39] and OTA [40] were used at concentrations of 0.01, 0.1, and 5 mg/L. The LOD was determined using a signal-to-noise ratio of 3:1, while the LOQ was calculated with a signal-to-noise ratio of 10:1.

### 2.5. Statistical Analysis

Statistical analyses were performed with StatGraphics Centurion XVII V.17.2.04 software (Statpoint Technologies Inc., Warrenton, VA, USA). The normality and homoscedasticity of the data were tested using the Shapiro–Wilk and Bartlett tests. All variables were analyzed via ANOVA, using Fisher’s LSD post hoc test to evaluate differences among groups. In all cases, the significance level was set at *p* < 0.05.

## 3. Results

### 3.1. Yeast Identification

The yeast strain KCB21_L2 was obtained from a kefir sample and identified as *P. fermentans* based on molecular analysis. A BLAST search of the NCBI database revealed that the sequence of isolated KCB21_L2 showed 100% identity with strain Y11A of *P. fermentans* (accession number MG478481.1). The sequence of isolated KCB21_L2 has been deposited in the European Nucleotide Archive (ENA) under the accession number PRJEB102054.

### 3.2. Ability of Pichia fermentans KCB21_L2 to Removal Aflatoxin B_1_, Fumonisin B_1_, and Ochratoxin A

Before evaluating removal capacity, the effect of mycotoxins on yeast growth and viability was examined. Yeast growth at pH values of 3.0, 5.5, and 7.0 was not affected by the presence of AFB_1_, FB_1_, and OTA (Table S4).

As shown in Table 1, the concentration of the three mycotoxins decreased after treatment with yeast at all pH levels tested. Significant differences were observed between the cell conditions, with VCs showing the greatest potential for removal at all pH levels for FB_1_ and at pH values of 5.5 and 7.0 for AFB_1_. *P. fermentans* KCB21_L2 showed higher AFB_1_ reduction at a pH of 3.0 (VCs = 92.44 ± 0.49%; HICs = 87.90 ± 4.26%). In the case of FB_1_, the pH did not affect the removal potential of the yeast, with removal values ranging from 72% to 88% for VCs and 49% to 64% for HICs.

In contrast, no statistically significant differences in OTA removal were found when analyzing the effects of either the cellular status (VCs and HICs) or pH levels (3.0, 5.5, and 7.0). OTA reduction percentages remained above 80% in all cases.

### 3.3. Effect of High Concentrations of Mycotoxins on Cell Viability and Removal Capacity of Pichia fermentans KCB21_L2

The viability of *P. fermentans* KCB21_L2 when exposed to high concentrations of AFB_1_ (20, 100 and 200 μg/L), FB_1_ (200, 1000 and 2000 μg/L) and OTA (1, 10 and 100 μg/L) was evaluated to assess its removal potential. In most cases, high concentrations of mycotoxins at the different pH values tested did not affect yeast growth. However, exposure to 2000 μg/L of FB_1_ resulted in a 30% reduction in cell growth at pH values of 5.5 and 7.0 (Table S5).

The removal capacity of *P. fermentans* KCB21_L2 against AFB_1_, FB_1_, and OTA was influenced by mycotoxin concentration and pH. In the case of AFB_1_, the highest reduction percentage occurred at the lowest concentration (20 μg/L). At the intermediate concentration (100 μg/L), pH strongly influenced the response, with removal decreasing as pH increased, and no effect was detected at a pH of 7.0. At the highest concentration (200 μg/L), the maximum reduction was observed at a pH of 3.0. Overall, AFB_1_ removal was more effective under acidic conditions (Table 2).

At a pH of 3.0, no reduction in FB_1_ was detected at any of the concentrations tested. At a pH of 5.5, significant reductions occurred at low and intermediate concentrations, whereas the highest reduction percentages were found at a pH of 7.0.

Finally, *P. fermentans* KCB21_L2 exhibited a high capacity to remove OTA at all concentrations and pH values tested. At the highest concentration, removal remained above 80% across all pH conditions. Both at this and the intermediate concentration (10 μg/L), reduction levels were consistently greater at pH values above 3.0. In the case of the lowest concentration (1 μg/L), reduction rates remained above 60% at all pH levels, with the highest value recorded at a pH of 7.0 (Table 2).

The results showed that *P. fermentans* KCB21_L2 tolerated high mycotoxin concentrations while maintaining viability and removal capacity. For transcriptomic analysis, the intermediate FB_1_ concentration (1000 μg/L) and the highest AFB_1_ (200 μg/L) and OTA (100 μg/L) concentrations were selected. A pH of 5.5 was selected to standardize the experimental conditions, as it is reported to be optimal for yeast metabolism and viability [41,42].

### 3.4. Effect of High Concentrations of AFB_1_, FB_1_, and OTA on the Transcriptome of Pichia fermentans KCB21_L2

**Figure 1:**
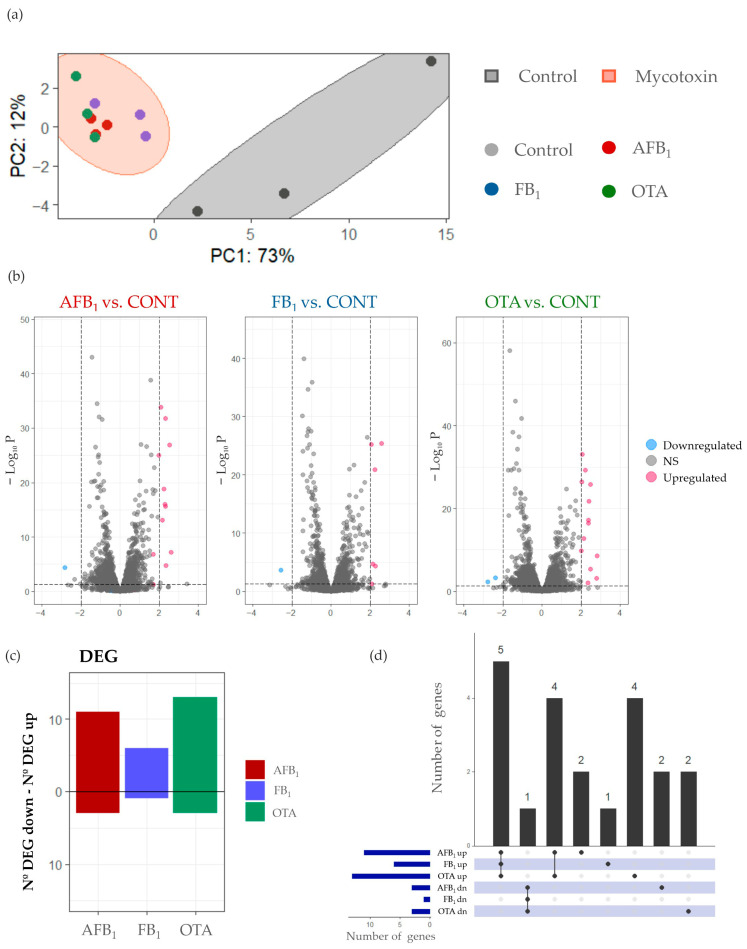
Transcriptomic analysis of *P. fermentans* KCB21_L2 at pH 5.5 under exposure to AFB_1_ (200 μg/L, red), FB_1_ (1000 μg/L, blue), and OTA (100 μg/L, green). (**a**) PCA plot samples exposed to mycotoxins (red shadow) and control samples without mycotoxins (gray). (**b**) Volcano plots showing upregulated DEGs (red) and downregulated DEGs (blue) in each treatment with respect to the control. Genes with non-significant changes in expression are shown in gray. (**c**) Total DEGs after exposure to AFB_1_ (red), FB_1_ (blue), and OTA (green), with upregulated genes labeled “up” and downregulated genes labeled “down.” (**d**) An UpSet plot showing shared and unique DEGs across mycotoxin treatments. Connected dots represent the common DEGs that were found between mycotoxin conditions.

PCA using transcriptomic data from controls and samples treated by mycotoxins (AFB_1_, FB_1_, OTA) (Figure 1a) revealed a clear separation between the control (gray) and treated (red) groups, with PC1 and PC2 explaining 73% and 12% of the total variability, respectively (85% overall). Replicates showed high consistency, with correlation coefficients above 0.94, confirming sample quality and experimental reproducibility.

The gene expressions in *P. fermentans* KCB21_L2 cultures exposed to each mycotoxin were compared with that in control. Out of the 5883 genes, only a small subset met the criteria for differential expression (|log_2_FC| ≥ 2; *p* < 0.05). Upregulated and downregulated genes for each mycotoxin treatment are shown in Figure 1b. After 12 h of exposure, AFB_1_ induced the expression of 11 genes and the repression of 3 and FB_1_ induced the upregulation of 6 genes and downregulation of one, whereas in the presence of OTA, 13 and 3 genes were up- and downregulated, respectively (Figure 1c).

An UpSet plot was generated to identify common and unique DEGs in the transcriptome of the yeast across the three mycotoxin treatments (Figure 1d). In total, five upregulated genes and one downregulated one were shared in the presence of the three mycotoxins tested. In addition, four upregulated genes were common after exposure to AFB_1_ and OTA. The remaining genes exhibited distinct expression patterns for each mycotoxin treatment, as shown in Figure 1d.

Table 3 and Table 4 show the up- and downregulated DEGs, together with their functional annotations from the COG, KEGG, and Pfam databases and the log_2_FC values, compared to the control, which were obtained through transcriptomic analysis. For uncharacterized proteins, complementary bioinformatic analyses (BLASTP and InterPro) were applied to detect conserved domains and putative functions.

The upregulated DEGs in *P. fermentans* KCB21_L2, which were shared in the presence of AFB_1_, FB_1_, and OTA, were identified as *put4*, *jen1*, *urc1*, and *sps19* (Table 3). The gene *put4* encodes a permease for proline, alanine, and glycine transport across the cell membrane, whereas *jen1* encodes a carboxylic acid transporter for lactate, pyruvate, and acetate. *urc1* encodes a GTP cyclohydrolase, essential for tetrahydrobiopterin biosynthesis and amino acid hydroxylation, and *sps19* encodes a peroxisomal 2,4-dienoyl-CoA reductase, a key enzyme in fatty acid catabolism. Exposure to AFB_1_ resulted in the upregulation of *ady2*, a gene encoding a transporter of ammonium, acetate, formate, and propionate, as well as an increased expression of *bet4*, which encodes the α-subunit of geranylgeranyl transferase that is an enzyme associated with intracellular vesicular trafficking. Under OTA treatment, an increased expression of *pox1*, which encodes an acyl-CoA oxidase involved in fatty acid metabolism, was detected.

In general, the presence of mycotoxins caused an increase in the activity of transmembrane transporters in the yeast *P. fermentans* KCB21_L2. In addition, exposure to AFB_1_ and OTA seemed to be associated with lipid peroxidation and amino acid metabolism, probably as a strategy to increase cellular energy and mitigate oxidative stress.

Considering downregulated genes, in all mycotoxin treatments, *P. fermentans* KCB21_L2 showed reduced expression of *por1*, which is a mitochondrial porin linked to ion transport and metabolism (Table 4). The *aro1* gene, encoding phenylpyruvate decarboxylase, was repressed under the AFB_1_ condition, whereas *rms1*, which encodes an N-lysine methyltransferase, was downregulated in the presence of OTA. These patterns indicate metabolic imbalance and cellular stress, which may contribute to senescence in the presence of mycotoxins.

**Figure 2:**
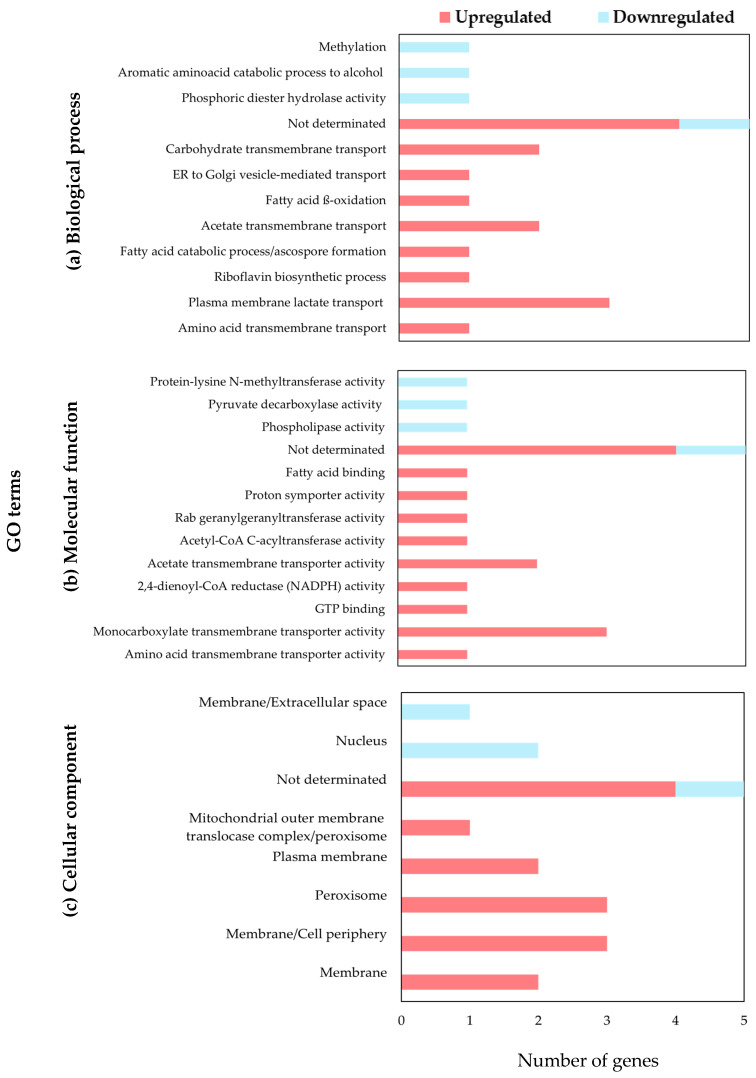
Functional annotation of DEGs in *P. fermentans* KCB21_L2 after exposure to AFB_1_, FB_1_, and OTA based on Gene Ontology (GO). DEGs are classified into (**a**) biological processes, (**b**) molecular functions, and (**c**) cellular components. Bars represent the number of genes assigned to each GO term; red indicates upregulated genes, and blue indicates downregulated ones.

A Gene Ontology (GO) functional analysis was performed to explore the roles of DEGs in *P. fermentans* KCB21_L2. Genes were annotated with biological processes, molecular function, and cellular components using information from the GO database. As shown in Figure 2a, upregulated DEGs associated with biological processes were primarily linked to vesicle and membrane transport activities. Additional processes included fatty acid catabolism and riboflavin biosynthesis, as well as methylation, phosphodiester hydrolase activity, and amino acid catabolism. Five DEGs could not be assigned to any specific biological process.

In terms of molecular functions, DEGs were predominantly associated with transmembrane transport, enzymatic activity, and molecular modification (Figure 2b). Transport-associated DEGs corresponded to amino acid, monocarboxylate, and proton transporters, which are essential for maintaining cellular homeostasis. Affected enzymatic activities included 2,4-dienoyl-CoA reductase, phospholipases, pyruvate decarboxylases, and acetyl-CoA C-acyltransferases, which are critical for the metabolism of fatty acids, phospholipids, and carbohydrates. Altered molecular modification functions included phospholipase activity, pyruvate carboxylase activity, and lysine N-methyltransferase activity, highlighting effects on central metabolism and post-translational modifications.

In the cellular compartment category, overexpressed DEGs were mainly associated with the peripheral/cell membrane, the plasma membrane, and peroxisomes, suggesting changes in transmembrane transport and cellular metabolism (Figure 2c). We also identified genes linked to the mitochondrial and peroxisomal outer membrane translocase complex, important for protein and metabolite transport. Repressed DEGs were primarily associated with the nucleus and extracellular regions, indicating a potential reduction in transcriptional regulation and extracellular interactions.

**Figure 3:**
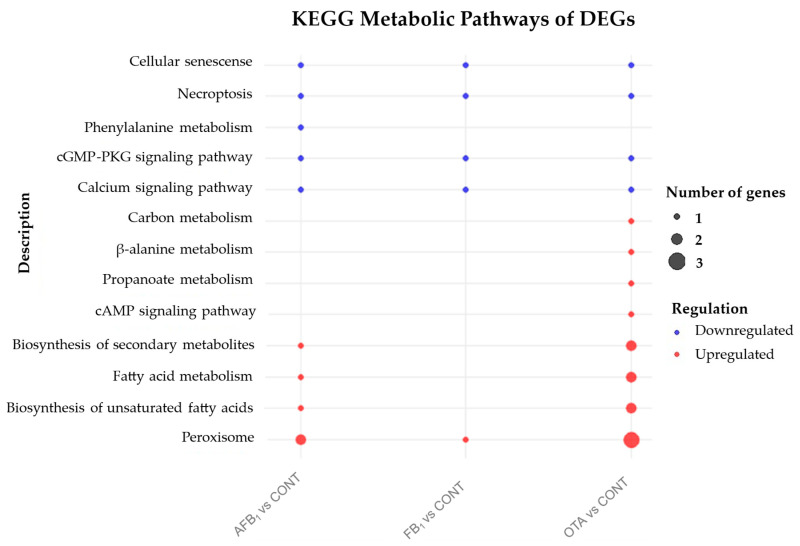
Descriptive representation of KEGG pathways associated with DEGs in *P. fermentans* KCB21_L2 after exposure to AFB_1_, FB_1_, and OTA. Pathways linked to upregulated DEGs are shown in red, whereas those linked to downregulated DEGs are shown in blue. Circle size reflects the relative significance of each pathway.

Additionally, the KEGG tool was used to integrate DEGs into specific metabolic pathways. As shown in Figure 3, after exposure to AFB_1_, FB_1_, and OTA, the upregulated DEGs were significantly enriched in the KEGG peroxisome pathway (ko04146). This pathway is associated with the activity of the enzyme 2,4-dienoyl-CoA reductase, which is essential for the β-oxidation of unsaturated fatty acids. In the transcriptome obtained after exposure to AFB_1_ and OTA, DEGs also showed significant enrichment in KEGG pathways related to unsaturated fatty acid biosynthesis (ko01040), fatty acid metabolism (ko01212), and secondary metabolism biosynthesis (ko01110). These pathways are associated with the activity of the enzymes acyl-CoA oxidase and acetyl-CoA acyltransferase. In addition, in the case of OTA treatment, other metabolic pathways were affected, including the cAMP signaling pathway (ko04024), propionate metabolism (ko00640), β-alanine metabolism (ko00410), and carbon metabolism (ko01200). These results suggest that the yeast adapts to mycotoxin stress through the reorganization of pathways involved in fatty acid turnover, amino acid degradation, and intracellular trafficking.

Among the downregulated DEGs, four KEGG pathways were significantly enriched, related to cellular senescence (ko04218), necroptosis (ko04217), cGMP-PKG signaling (ko04022), and calcium signaling (ko04020). These results suggest a negative impact on cell viability and function due to the accumulation of damaged cells and alterations in cell homeostasis.

The results of the degradation analyses showed no detectable mycotoxin metabolites, suggesting that *P. fermentans* KCB21_L2 may reduce through mechanisms other than direct degradation, possibly involving an unknown pathway.

## 4. Discussion

The search for innovative strategies to control mycotoxin contamination remains a global priority, given the significant threat they pose to food safety and public health. Among the available approaches, the use of microorganisms isolated from food capable of removing mycotoxins stands out as one of the most promising alternatives, not only due to its effectiveness but also because of its potential safety in food systems [12,13]. Therefore, understanding the mechanisms underlying microbial reduction is essential to support its future application. In this study, the potential of yeast *P. fermentans* KCB21_L2, isolated from kefir, to remove AFB_1_, FB_1_, and OTA was evaluated, and analysis identified the mechanisms involved in detoxification from a transcriptomic perspective. To our knowledge, this is the first study to evaluate this species as a potential mycotoxin-removing agent.

Biological decontamination of mycotoxins by microorganisms occurs mainly through biodegradation and/or adsorption into their cell walls [15]. To determine the mechanism involved in the detoxification of AFB_1_, FB_1_, and OTA by *P. fermentans* KCB21_L2, assays were performed using viable cells (VCs) and heat-inactivated cells (HICs). Adsorption is a physical removal process which is independent of the physiological state of cells; therefore, it can also occur in non-viable cells [13]. Moreover, several studies have reported that heat treatment may enhance detoxification by adsorption by inducing structural alterations in the microbial cell wall [43]. In this study, a significant reduction in AFB_1_, FB_1_, and OTA was observed in both VCs and HICs, indicating that adsorption plays a key role in the removal of these mycotoxins. It is known that pH is a relevant factor that affects mycotoxin adsorption, especially in the case of OTA. The binding of this toxin to yeast walls is enhanced by acidic conditions (pH < 4.0) [43,44], whereas alkaline conditions decrease OTA–yeast affinity due to the ionization and destabilization of the three-dimensional β-glucan structure [43,45]. Although they are less extensively investigated, acidic conditions have also been reported to enhance FB_1_ removal by yeast, mainly due to cell wall disorganization and the stabilization of acidic groups that promote interactions with cellular components [46]. In contrast with these findings, our results showed that acidic pH did not lead to higher reduction percentages for either OTA or FB_1_. However, *P. fermentans* KC21_L2 showed increased AFB_1_ removal at a pH of 3.0, in agreement with previous studies that link this effect to partial denaturation of surface proteins under acidic conditions, which exposes additional binding sites and facilitates AFB_1_ binding [45,47].

In addition to pH, another key factor influencing microbial decontamination is the concentration of mycotoxins, since high levels could induce cytotoxic effects in removing microorganisms, affecting their viability and metabolic activity [48]. In this study, a significant reduction in the viability of *P. fermentans* KCB21_L2 was observed only at the highest concentration of FB_1_ tested (2000 μg/L), indicating that the yeast could be applied even in foods contaminated with levels higher than twice the maximum permitted by the EU [49]. In addition to direct effects on cellular viability, the concentration used also directly impacts removal capacity, especially when the adsorption process is involved [45]. Some studies have shown that OTA adsorption is not linear but is highly efficient at low concentrations, tending to stabilize as the mycotoxin concentration increases because of the progressive saturation of the binding sites on the yeast cell wall [45]. This phenomenon has also been observed with other mycotoxins, such as AFB_1_ and FB_1_ [50,51]. In view of this, the results of reduction in high concentrations of AFB_1_ appear to show saturation of binding sites between 20 μg/L and 100 μg/L, especially at acidic pH values. This indicates that the binding sites on the yeast cell wall were fully occupied by AFB_1_ under these conditions, limiting the effectiveness of removal at higher concentrations. In the case of OTA, removal did not exhibit saturation even at the highest concentration tested (100 μg/L), indicating that the interaction between OTA and the yeast remained effective at that concentration. For FB_1_, saturation was observed at 1000 μg/L under acidic conditions, whereas maximum reduction was achieved in 2000 μg/L under neutral pH, highlighting the strong influence of pH on binding capacity and removal efficiency. In summary, these findings highlight the importance of considering both toxin concentration and pH when designing biological decontamination strategies, as different mycotoxins exhibit different behaviors and may require specific conditions for optimal removal [52].

Although adsorption seems to be related to mycotoxin decontamination via *P. fermentans* KCB21_L2, VCs are capable of removing AFB_1_ and FB_1_ more efficiently than HICs, suggesting the involvement of additional active mechanisms. Previous studies have shown that fungal and yeast enzymes, such as laccase from *Trametes versicolor*, manganese peroxidase from *Pleurotus ostreatus*, and *Candida versatilis* can degrade AFB_1_ at acidic pH (4.0–5.0) [53,54,55,56]. Regarding FB_1_, carboxylesterase enzymes such as FumD and FumDSB, originally isolated from microorganisms, have been reported to effectively degrade this mycotoxin across a broad pH range of 5.0 to 9.0 [57,58]. These findings are consistent with our study, in which the highest reduction occurred at similar pH values, suggesting the possible involvement of enzymatic activity.

Considering the possible active mechanisms involved in mycotoxin removal, it is essential to investigate the molecular and cellular responses of *P. fermentans* KCB21_L2 to mycotoxin exposure to elucidate its putative removal pathways. Transcriptional analysis revealed a low number of differentially expressed genes (DEGs) in the yeast response to exposure to each mycotoxin, which differed from similar studies conducted with other microorganisms [59,60,61]. This difference can be attributed to the fact that many of these studies used higher concentrations of mycotoxins even when cell viability was affected [60]. Furthermore, most published studies use less stringent statistical criteria, such as |log_2_FC| ≥ 1 or even |log_2_FC| ≥ 0.5, which could lead to an overestimation of the number of genes identified as DEGs [61,62,63]. In contrast, we used a stricter threshold of |log_2_FC| ≥ 2, which reduced the number of genes detected but allowed the identification of those with more pronounced and biologically relevant expression changes.

In yeasts such as *Apiotrichum mycotoxinivorans*, exposure to high OTA concentrations is known to induce a cellular response associated with oxidative stress, which reduces toxicity [60]. Although the expression of antioxidant-related genes was not modified in *P. fermentans* KCB21_L2, exposure to mycotoxins induced overexpression of peroxisomal genes, which play a central role in stress responses through metabolic processes such as fatty acid β-oxidation [62]. Specifically, *sps19* was upregulated by all three mycotoxins, *pot1* by AFB_1_ and OTA, and *pox1* by OTA, a pattern consistent with oxidative stress responses in *S. cerevisiae* [64]. These genes encode enzymes involved in different stages of β-oxidation: *pox1* catalyzes acyl-CoA oxidation, *sps19* facilitates degradation of unsaturated fatty acids, and *pot1* releases acetyl-CoA [65]. Their activation suggests that *P. fermentans* KCB21_L2 may utilize lipid metabolism to generate energy in response to mycotoxin-induced stress.

Exposure to mycotoxins also affects protein-level responses in yeasts such as *A. mycotoxinivorans* [60]. The presence of the three mycotoxins in *P. fermentans* KCB21_L2 tested in the present work induced overexpression of *put4*, a proline permease gene that potentially enhances proline uptake and stress tolerance through its antioxidant and osmoprotective functions [66]. The repression of *aro10* in response to AFB_1_ suggests a metabolic shift to prioritize essential survival pathways over aromatic amino acid catabolism [67]. In contrast, upregulation of *bet4* may reflect increased Rab protein prenylation and vesicular trafficking [68]. When the transcriptome of the yeast was analyzed after FB_1_ treatment, an overexpression of the gene *urc1* (which encodes a GTP cyclohydrolase) was observed, but BLASTP analysis revealed no similarity to known hydrolases responsible for the degradation [59]. This suggests that *urc1* upregulation may reflect enhanced uracil catabolism under mycotoxin-induced stress, helping recover nitrogen and generate essential nucleotides [69]. *por1* was the only gene consistently downregulated across all three treatments. Broeskamp et al. [70] reported that the loss of this gene reduces autophagic capacity, disrupts vacuolar and lipid homeostasis, and increases cellular susceptibility to stress and death. According to this, the observed decrease in *por1* expression may promote intracellular accumulation of mycotoxins and contribute to the overexpression of lipid peroxidation-related genes such as *sps19* and *pox1*.

Mycotoxin exposure also led to a marked upregulation of sugar transporter genes in *P. fermentans* KCB21_L2, particularly those of the major facilitator superfamily (MFS), including *jen1* [71]. These transporters have been reported to mediate the efflux of drugs and xenobiotics, thereby reducing cellular toxicity [72]. This adaptive response to mycotoxins is not specific to *P. fermentans* KCB21_L2, as transcriptomic studies in *Y. lipolytica* and *S. cerevisiae* have similarly shown MFS overexpression in the presence of citrinin and OTA [73,74]. In addition, several studies have reported that some genes related to MFS transporters such as *jen1* and *ady2* are induced in the presence of lactic acid [75,76]. This organic acid is mainly produced by lactic acid bacteria, but some strains of *P. fermentans* can also synthesize it [77]. It has been reported that lactic acid can reduce AFB_1_ to less toxic metabolites, such as AFB_2_ [78], which could also be related to the reduction potential of the yeast under study.

Although adsorption is a key mechanism in removal, transcriptional analysis of *P. fermentans* KCB21_L2 did not show significant changes in genes related to β-glucan or mannoprotein biosynthesis after exposure to mycotoxins. These results contrast with those reported by Oporto et al. [79], who observed that exposure to patulin increased the expression of mannoprotein genes in *S. cerevisiae*.

On the order hand, no upregulation was observed in genes encoding enzymes previously implicated in mycotoxin degradation, including carboxypeptidases A and Y with OTA [60], glycerol dehydrogenase with AFB_1_ [61], and alpha/beta hydrolases, esterases, and transferases with FB_1_ [59]. Likewise, the analyses did not reveal typical degradation products of any of the mycotoxins under the experimental conditions used. In addition to intrinsic factors such as the initial concentration of mycotoxins or pH, extrinsic factors such as incubation time have a considerable influence on the decontamination mechanism [80]. While adsorption occurs rapidly within minutes [81], microbial biodegradation requires longer periods, and its efficiency varies among strains. For instance, *Bacillus amyloliquefaciens* ZG08 achieved 81% degradation of AFB_1_ after 72 h, whereas *Pseudomonas putida* reduced 90% of AFB_1_ within 24 h [61,82]. In the case of OTA, *A. mycotoxinivorans* reached 95% degradation after 24 h [60], which could explain the low detection of degradation metabolites after 12 h of incubation.

The UHPLC-Q-TOF-MS analysis focused on previously reported metabolites, meaning that the absence of detection does not exclude the formation of new compounds or those present at levels below the detection limit. This represents a common limitation in mycotoxin studies, given the difficulty of identifying masked or unknown degradation products [83]. Recent studies using isotopic labelling strategies have identified new AFB_1_ degradation metabolites [84], which were not included in our UHPLC-Q-TOF-MS analysis and may therefore also explain their absence in the results.

Although transcriptomic analysis did not reveal any DEGs associated with mycotoxin degradation or degradation products, the results show that CV eliminated a greater number of mycotoxins than CIT. In this context, Halon et al. [85] showed that only a minority of degradation genes are inducible in whiteflies, with most expressed constitutively. Similarly, Alberts et al. [86] reported that AFB_1_ degradation in *Rhodococcus erythropolis* was mediated by non-identified extracellular enzymes expressed constitutively. Therefore, the active elimination capacity of *P. fermentans* KCB21_L2 may also have involved a constitutive and non-inducible mechanism, although further studies are needed to confirm this.

Some authors have suggested that mycotoxin removal in microorganisms is likely the result of a combination of mechanisms, including stress responses, secretion of extracellular compounds, and adsorption into the cell wall [74,87,88]. In this regard, the elimination of mycotoxins in *P. fermentans* KCB21_L2 appears to be a complex and multifactorial process involving both cell wall adsorption and an active mechanism that is variable and difficult to determine.

## 5. Conclusions

This study provides information on the possible mechanisms used by *P. fermentans* KCB21_L2 to remove AFB_1_, FB_1_, and OTA. The results suggest that adsorption into the cell wall is the main mechanism, as similar elimination percentages were observed between viable and thermally inactivated cells, especially in the case of OTA. However, the greater capacity for removing AFB_1_ and FB_1_ in viable cells also indicates the possible involvement of active processes. Exposure to high mycotoxin concentrations induced a cellular response marked by changes in primary metabolism, including the activation of genes related to amino acid catabolism, fatty acid metabolism, and membrane transport. In summary, mycotoxin removal in *P. fermentans* KCB21_L2 integrates both adsorption processes and active mechanisms that are not inducible by the presence of mycotoxins, although determining the precise mechanisms remains complex and represents a limitation of this study.

## Figures and Tables

**Table 1:** Percentages of the reduction in each mycotoxin in viable cells (VCs) and heat-inactivated cells (HICs) of *P. fermentans* KCB21_L2 at different pH values. The results are the mean ± standard deviation of two replicates. Comparisons were independently made for each mycotoxin. For each mycotoxin (AFB_1_, FB_1_, and OTA), different letters (a–c) within the same row (pH) indicate significant differences (*p* ≤ 0.05) among the control, VCs, and HICs. The 0% value in the control corresponds to the initial concentration of each mycotoxin, indicating that no detoxification occurred in the absence of cells. For each cell condition (VCs and HICs), different letters (Y–Z) in the same column (mycotoxin) indicate significant differences (*p* ≤ 0.05) among the percentages of reduction obtained at each pH (3.0, 5.5, and 7.0). The absence of a letter indicates no statistically significant differences.

Mycotoxin Reduction (%)				
pH	Treatment	AFB_1_	FB_1_	OTA
3.0	Control	0 a	0 a	0 a
	VC	92.44 ± 0.49 bY	75.72 ± 6.52 c	97.15 ± 0.16 b
	HIC	87.90 ± 4.26 bY	53.24 ± 3.56 b	96.81 ± 0.35 b
5.5	Control	0 a	0 a	0 a
	VC	86.25 ± 0.63 cZY	88.06 ± 1.30 c	96.44 ± 1.61 b
	HIC	75.18 ± 5.78 bZY	63.94 ± 6.46 b	94.19 ± 3.34 b
7.0	Control	0 a	0 a	0 a
	VC	76.39 ± 4.31 cZ	72.11 ± 4.14 c	89.48 ± 6.52 b
	HIC	59.01 ± 5.12 bZ	49.22 ± 0.76 b	84.22 ± 12.48 b

**Table 2:** Percentages of reduction in each mycotoxin (AFB_1_, FB_1_, and OTA) after exposure to *P. fermentans* KCB21_L2 at different pH levels (3.0, 5.5, and 7.0). Results are the mean ± standard deviation of two samples. The 0% value in the control corresponds to the initial concentration of each mycotoxin, indicating that no detoxification occurred in the absence of cells. Asterisks indicate the level of statistical significance compared to the control: *p* ≤ 0.05 (*); *p* ≤ 0.005 (**); and *p* ≤ 0.0005 (***).

Mycotoxin Reduction (%)				
		pH 3.0	pH 5.5	pH 7.0
Control		0	0	0
AFB_1_	200 µg/L	64.10 ± 2.70 **	26.91 ± 1.17 **	37.37 ± 7.01 *
	100 µg/L	87.87 ± 0.54 ***	53.81 ± 10.44 *	2.96 ± 0.38
	20 µg/L	> 95 ± 0.43 **	93.98 ± 6.02 ***	93.43 ± 0.92 **
FB_1_	2000 µg/L	0	12.62 ± 9.87	55.20 ± 4.03 *
	1000 µg/L	0	41.43 ± 5.47 *	41.66 ± 2.16 **
	200 µg/L	26.08 ± 8.94	26.90 ± 4.22 *	39.82 ± 1.18 *
OTA	100 µg/L	92.35 ± 0.24 ***	83.14 ± 4.54 *	85.62 ± 4.74 *
	10 µg/L	91.06 ± 0.08 **	60.23 ± 0.49 ***	69.36 ± 7.23 *
	1 µg/L	70.69 ± 6.64 *	60.60 ± 7.5 *	85.65 ± 1.17 **

**Table 3:** Protein prediction based on upregulated DEGs in *P. fermentans* KCB21_L2 after exposure to AFB_1_ (200 μg/L), FB_1_ (1000 μg/L) and OTA (100 μg/L). The gene identification code (gene ID), expression level (log_2_FC) with AFB_1_, FB_1_, and OTA, protein prediction, homologous gene name in *Saccharomyces*, COG category, and KEGG pathway are described.

Upregulated Genes							
Gene ID	log_2_FC			Pfam Domain	Gene Name	COG	KEGG Pathway/Possible Function
	AFB_1_	FB_1_	OTA				
g1490	2.312	2.068	2.220	Amino acid permease	*put4*	Amino acid metabolism and transport	Uncharacterized/Proline transport
g1812	2.346	2.281	2.480	Carboxylic acid transporter	*jen1*	Amino acids, carbohydrates and inorganic ions metabolism and transport	Uncharacterized/Lactate transport
g2113	2.239	2.594	2.400	GTP cyclohydrolase	*urc1*	Coenzyme metabolism and transport	Uncharacterized/Uracil catabolism
g3629	2.538	2.243	2.484	Peroxisomal 2,4-dienoyl-CoA reductase	*sps19*	Biosynthesis, degradation and transport processes in secondary metabolism	Peroxisome (ko04146)/β-oxidation of fatty acids
g5372	2.608	2.132	2.825	Carboxylic acid transporter	*jen1*	Amino acids, carbohydrates, and inorganic ions metabolism and transport	Uncharacterized/Lactate transport
g4171	2.304		2.378	Ammonium transporter	*ady2*	Function unknown	Uncharacterized/Ammonium transport
g5373	2.167		2.132	Carboxylic acid transporter	*jen1*	Amino acids, carbohydrates and inorganic ions metabolism and transport	Uncharacterized/Lactate transport
g5556	2.087		2.058	Acetyl-CoA acyltransferase	*pot1*	Lipids metabolism and transport	Peroxisome (ko04146); Biosynthesis of unsaturated fatty acids (ko01040) and secondary metabolites (ko01110); Fatty acid metabolism (ko01212)/
g5835	2.331		2.386	Ammonium transporter	*ady2*	Function unknown	Uncharacterized/Ammonium transport
g3716	3.416			DNA-binding domain of transposase	-	Function unknown	Uncharacterized
g5755	2.050			Geranylgeranyl transferase type-2 alpha subunit	*bet4*	Post-translational modification, replacement of proteins and chaperones	Uncharacterized/Post-translational modification of proteins
g1403			2.001	Sugar transporter	-	Inorganic ions metabolism and transport	Uncharacterized
g1640			2.790	Uncharacterized protein	-	-	-
g2207			2.028	Belonging to the acyl-CoA oxidase family	*pox1*	Lipids metabolism and transport	Peroxisome (ko04146); Biosynthesis of unsaturated fatty acids (ko01040) and secondary metabolites (ko01110); Fatty acid metabolism (ko01212); cAMP signaling pathway (ko04024); Propanoate metabolism (ko00640). ß-alanine (ko00410) and carbon (k001200)
g3754			2.340	Uncharacterized protein	-	-	-
g5627		2.083		Uncharacterized protein	-	-	-

**Table 4:** Protein prediction based on downregulated DEGs in *P. fermentans* KCB21_L2 after exposure to AFB_1_ (200 μg/L), FB_1_ (1000 μg/L) and OTA (100 μg/L). The gene identification code (gene ID), expression level (log_2_FC) in AFB_1_, FB_1_, and OTA, protein prediction, homologous gene name in *Saccharomyces*, COG category, and KEGG pathway are described.

Downregulated Genes							
Gene ID	log_2_FC			Pfam Domain	Gene Name	COG	KEGG Pathway/Possible Function
	AFB_1_	FB_1_	OTA				
g5397	−2.839	−2.571	−2.372	Porin	*por1*	Inorganic ions metabolism and transport	Cellular senescence (ko04218); Necroptosis (ko04217); cGMP-PKG signaling pathway (ko04022); Calcium signaling pathway (ko04020)
g879	−2.046			Calcineurin-like phosphoesterase	-	Lipids metabolism and transport	Uncharacterized
g1851	−2.327			Phenylpyruvate decarboxylase	*aro10*	Amino acids and coenzymes metabolism and transport	Phenylalanine metabolism (ko00360)
g2603			−2.774	N-lysine methyltransferase SET7	*rms1*	Inorganic ions metabolism and transport	Uncharacterized/Monomethylation of 60S ribosomal protein L42
g5391			−5.115	Uncharacterized protein	-	-	-

## Data Availability

Raw sequencing data have been deposited in the European Nucleotide Archive at EMBL–EBI under the project name PRJEB102104. Further inquiries can be directed to the corresponding author.

## Supplementary Materials

The following supporting information can be downloaded at: https://www.mdpi.com/article/10.3390/foods14244181/s1. Table S1. Aflatoxin B_1_ degradation products were analyzed using UHPLC-Q-TOF-MS; Table S2. Fumonisin B_1_ degradation products were analyzed using UHPLC-Q-TOF-MS; Table S3. Ochratoxin A degradation products were analyzed using UHPLC-Q-TOF-MS; Table S4. Absorbance values at 630 nm after culturing *P. fermentans* KCB21_L2 on PDB at different pH and treatments; Table S5. Counts of viable cells of *P. fermentans* KCB21_L2 after exposure to high concentrations of mycotoxins, at different pH values.
