# Supplementary material for: Mycotoxin Removal and Transcriptional Response of Pichia fermentans KCB21_L2
Source: Foods. 2025 Dec 5;14(24):4181. doi: 10.3390/foods14244181 (PMC12732294; doi:10.3390/foods14244181)
Supplement: Supplementary file 1 [file foods-14-04181-s001.zip › foods-3941277-supplementary.pdf]

Table S1. Aflatoxin B_1 degradation products analyzed using UHPLC-Q-TOF-MS. The table shows the compound names and their corresponding molecular formulas.

Aflatoxin Degradation Products	Molecular Formula
1,2,5a,8a-tetrahydro-10-H-cyclobuta[c]furo[3′,2′:4,5]furo[2,3-h]chromen-10-one	C15H11O4
1,2-dihydroxy-5-(4-hydroxy-2-oxo-2,3,3a,8a-tetrahydrofuro[2,3-b]benzofuran-5-yl)cyclopentanecarboxylic acid	C16H17O8
1-hydroxy-2-(4-hydroxy-6-methoxy-2-oxo-2,3,3a,8a-tetrahydrofuro[2,3-b]benzofuran-5-yl)-5-oxocyclopentanecarboxylic acid	C17H17O9
1-hydroxy-2-oxo-5-(2,4,6-trihydroxy-2,3,3a,8a-tetrahydrofuro[2,3-b]benzofuran-5-yl)cyclopentanecarboxylic acid	C16H17O9
2-(2,4-dihydroxy-2,3,3a,8a-tetrahydrofuro[2,3-b]benzofuran-5-yl)-5-hydroxycyclopent-1-enecarboxylic acid	C16H17O7
2,3,3a,4,5,8a-hexahydrofuro[2,3-b]benzofuran-2,6-diol	C10H13O4
2,3,3a,6a-tetrahydrofuro[2,3-b]furan 8-methoxy-1,2-dihydro-3H-cyclopenta[b]benzofuran-3-one	C16H12O5
2,3,6a,9a-tetrahydro-4-methoxycyclopenta[c]furo[2,3:4-5]furo[2,3-h]chromene-1,11-dione	C17H13O6
3-(hydrometyl)-8,9-dihydro-2H-furo[2,3-h]chromen-8-ol	C12H14O4
3a,4,5,8a-tetrahydrofuro[2,3-b]benzofuran-2,6-diol	C10H11O4
8,9-Dihydroxy-8,9-dihydro-aflatoxin B_1	C17H14O8
8-Dichloroacetaxy-8,9-dihydro-aflatoxin B_1	C19H14Cl2O8
Aflatoxicol	C17H14O6
Aflatoxin B_1-8,9-epoxide	C17H12O7
Aflatoxin B_{2a}	C17H14O7
Aflatoxin D_1	C16H14O5
Aflatoxin D_2	C11H10O4
Aflatoxin P_1	C16H10O6
Phthalic anhydride	C8H4O3
Unnamed AFB_1 degraded compound 1	C16H14O6
Unnamed AFB_1 degraded compound 2	C14H11O6
Unnamed AFB_1 degraded compound 3	C16H13O5
Unnamed AFB_1 degraded compound 4	C17H15O6
Unnamed AFB_1 degraded compound 5	C18H33N3O3
Unnamed AFB_1 degraded compound 6	C18H34N3O3
Unnamed AFB_1 degraded compound 7	C6H10N
Unnamed AFB_1 degraded compound 8	C6H12NO
Unnamed AFB_1 degraded compound 9	C6H7
Unnamed AFB_1 degraded compound 10	C17H11O7
Unnamed AFB_1 degraded compound 11	C16H12O7
Unnamed AFB_1 degraded compound 12	C17H23O9
Unnamed AFB_1 degraded compound 13	C16H13O7
Unnamed AFB_1 degraded compound 14	C16H17O6
Unnamed AFB_1 degraded compound 15	C17H15O8
Unnamed AFB_1 degraded compound 16	C16H15O7

	Unnamed AFB_1 degraded compound 17	C12H8O4
	Unnamed AFB_1 degraded compound 18	C13H12O2
	Unnamed AFB_1 degraded compound 19	C14H10O4
	Unnamed AFB_1 degraded compound 20	C14H12O3
	Unnamed AFB_1 degraded compound 21	C14H16O2
	Unnamed AFB_1 degraded compound 22	C16H16O6
	Unnamed AFB_1 degraded compound 23	C14H20O2
	Unnamed AFB_1 degraded compound 24	C16H12O2
	Unnamed AFB_1 degraded compound 25	C16H18O5
	Unnamed AFB_1 degraded compound 26	C16H20O4
	Unnamed AFB_1 degraded compound 27	C17H20O7
	Unnamed AFB_1 degraded compound 28	C18H16O8
	Unnamed AFB_1 degraded compound 29	C6H10
	Unnamed AFB_1 degraded compound 30	C6H12O
	Unnamed AFB_1 degraded compound 31	C8H12O
	Unnamed AFB_1 degraded compound 32	C9H10O3
	Unnamed AFB_1 degraded compound 33	C14H12O5
	Unnamed AFB_1 degraded compound 34	C9H14O2
	Unnamed AFB_1 degraded compound 35	C9H14O4
	Unnamed AFB_1 degraded compound 36	C9H18O2
	Unnamed AFB_1 degraded compound 37	C16H16O7
	Unnamed AFB_1 degraded compound 38	C17H20O9
	Unnamed AFB_1 degraded compound 39	C17H12O8
	Unnamed AFB_1 degraded compound 40	C17H8O8
	Unnamed AFB_1 degraded compound 41	C17H18O8
	Unnamed AFB_1 degraded compound 42	C17H14O5
	Unnamed AFB_1 degraded compound 43	C17H16O6
	Unnamed AFB_1 degraded compound 44	C14H10O6
	Unnamed AFB_1 degraded compound 45	C19H18O8
	Unnamed AFB_1 degraded compound 46	C12H21N2O
	Unnamed AFB_1 degraded compound 47	C12H22N2O2
	Unnamed AFB_1 degraded compound 48	C12H23N2O2
	Unnamed AFB_1 degraded compound 49	C13H10O2
	Unnamed AFB_1 degraded compound 50	C13H17O4
	Unnamed AFB_1 degraded compound 51	C14H13O5
	Unnamed AFB_1 degraded compound 52	C14H8O3
	Unnamed AFB_1 degraded compound 53	C15H10O3
	Unnamed AFB_1 degraded compound 54	C15H11O5
	Unnamed AFB_1 degraded compound 55	C15H11O7
	Unnamed AFB_1 degraded compound 56	C15H12O4
	Unnamed AFB_1 degraded compound 57	C15H13O7
	Unnamed AFB_1 degraded compound 58	C16H11O6
	Unnamed AFB_1 degraded compound 59	C16H12O4
	Unnamed AFB_1 degraded compound 60	C16H15O5
	Unnamed AFB_1 degraded compound 61	C17H13O7
	Unnamed AFB_1 degraded compound 62	C17H15O7
	Unnamed AFB_1 degraded compound 63	C17H16O7

Table S2. Fumonisin B_1 degradation products analyzed using UHPLC-Q-TOF-MS. The table shows the compound names and their corresponding molecular formulas.

**Fumonisin B_1 Degradation Products**	**Molecular Formula**
Hydrolyzed fumonisin B_1	C22H47NO5
Partial hydrolyzed fumonisin B_1	C28H53NO10
Fumonisin FP_1	C39H62NO16
Fumonisin C_1	C33H57NO15
Esterified FB_1 - linoleic acid	C52H89NO16
Esterified FB_1 - palmitic acid	C50H89NO16
Esterified FB_1 - oleic acid	C52H91NO16
N-(deossi-D-frutto-1yl)-fumonisin B_1	C40H69NO20
N-(carbossimetil)-fumonisin B_1	C36H61NO17
Lysil-fumonisin B_1	C43H75N3O17
Fumonisin B_1+Metossiglucosio	C50H85NO24
Heptadecanone	C17H34O
Isononadecene	C19H38
Octadecenal	C18H34O
Eicosane	C20H42

Table S3. Ochratoxin A degradation products analyzed using UHPLC-Q-TOF-MS. The table shows the compound names and their corresponding molecular formulas.

**Ochratoxin A Degradation Products**	**Molecular Formula**
4-Hydroxy ochratoxin A-β-D-glucoside	C26H28ClNO12
4-hydroxy-ochratoxin A methyl ester	C21H20ClNO7
Ethylamide ochratoxin A	C22H23ClN2O5
Hydroxy-ochratoxin A	C20H18ClNO7
Lactone-opened ochratoxin A	C20H20ClNO7
Lactone-opened ochratoxin B	C20H21NO7
Lactone-opened ochratoxin β	C11H9O5
Ochratoxin A cellobiose ester	C32H38ClNO16
Ochratoxin A decarboxylated	C19H18ClNO4
Ochratoxin A quinone	C20H17NO7
Ochratoxin B	C20H19NO6
Ochratoxin B ethyl ester	C22H23NO6
Ochratoxin B methyl ester	C21H21NO6
Ochratoxin C	C22H22ClNO6
Ochratoxin α	C11H9ClO5
Ochratoxin α methyl ester	C12H11ClO5
Ochratoxin α-amide	C11H12ClNO4
Ochratoxin β	C11H10O5

Table S4. Absorbance values at 630 nm after culturing *P. fermentans* KCB21_L2 on PDB at different pH levels (3.0, 5.5, and 7.0) and treatments (control, AFB_1 (10 μg/L), FB_1 (100 μg/L), and OTA (0.5 μg/L)). The results are the mean ± standard deviation of two replicates. For each pH value, comparisons were performed between different treatments. There were no significant differences between the control and any treatment ($p > 0.05$).

**pH**	**Treatment**			
	**Control**	**AFB_1**	**FB_1**	**OTA**
pH 3.0	1.38 ± 0.02	1.41 ± 0.03	1.44 ± 0.02	1.43 ± 0.02
pH 5.5	1.49 ± 0.04	1.50 ± 0.02	1.56 ± 0.03	1.51 ± 0.02
pH 7.0	1.52 ± 0.01	1.50 ± 0.03	1.50 ± 0.04	1.49 ± 0.01

Table S5. Counts of viable cells (CFU/mL) of *P. fermentans* KCB21_L2 after exposure to high concentrations of AFB_1, FB_1, and OTA, at different pH values (3.0, 5.5, and 7.0). The results are the mean ± standard deviation of two replicates. Asterisks (*) indicate significant differences ($p \leq 0.05$) compared to the control.

		**Count of viable cells (CFU/mL)**		
		**pH 3.0**	**pH 5.5**	**pH 7.0**
**Control**		$9.79 \times 10^7 \pm 2.10 \times 10^6$	$3.95 \times 10^8 \pm 5.00 \times 10^6$	$2.78 \times 10^8 \pm 1.02 \times 10^8$
**AFB_1**	200 μg/L	$8.80 \times 10^7 \pm 1.00 \times 10^6$	$2.05 \times 10^8 \pm 4.50 \times 10^7$	$1.40 \times 10^8 \pm 8.50 \times 10^6$
	100 μg/L	$7.95 \times 10^7 \pm 1.15 \times 10^7$	$3.30 \times 10^8 \pm 4.00 \times 10^7$	$2.15 \times 10^8 \pm 8.50 \times 10^7$
	20 μg/L	$6.55 \times 10^7 \pm 1.25 \times 10^7$	$3.89 \times 10^8 \pm 1.92 \times 10^8$	$1.66 \times 10^8 \pm 2.75 \times 10^7$
**FB_1**	2000 μg/L	$8.70 \times 10^7 \pm 3.00 \times 10^6$	$1.22 \times 10^8 \pm 1.50 \times 10^7$ *	$1.24 \times 10^8 \pm 2.60 \times 10^7$ *
	1000 μg/L	$8.10 \times 10^7 \pm 7.00 \times 10^6$	$1.51 \times 10^8 \pm 1.60 \times 10^7$	$1.97 \times 10^8 \pm 1.04 \times 10^8$
	200 μg/L	$8.30 \times 10^7 \pm 2.70 \times 10^7$	$2.80 \times 10^8 \pm 9.00 \times 10^7$	$3.55 \times 10^8 \pm 3.50 \times 10^7$
**OTA**	100 μg/L	$8.05 \times 10^7 \pm 5.50 \times 10^6$	$3.10 \times 10^8 \pm 3.00 \times 10^7$	$1.68 \times 10^8 \pm 2.50 \times 10^6$
	10 μg/L	$1.18 \times 10^8 \pm 1.65 \times 10^7$	$3.40 \times 10^8 \pm 2.00 \times 10^7$	$1.34 \times 10^8 \pm 6.50 \times 10^6$

1 μg/L	$9.05 \times 10^7 \pm 1.45 \times 10^7$	$3.30 \times 10^8 \pm 3.00 \times 10^7$	$2.35 \times 10^8 \pm 9.50 \times 10^7$
